# NF-κB/RelA controlled A20 limits TRAIL-induced apoptosis in pancreatic cancer

**DOI:** 10.1038/s41419-022-05535-9

**Published:** 2023-01-03

**Authors:** Claudia Geismann, Charlotte Hauser, Frauke Grohmann, Christian Schneeweis, Nico Bölter, Jan-Paul Gundlach, Günter Schneider, Christoph Röcken, Christian Meinhardt, Heiner Schäfer, Stefan Schreiber, Alexander Arlt

**Affiliations:** 1 Department of Internal Medicine I, Laboratory of Molecular Gastroenterology & Hepatology, UKSH-Campus Kiel, Kiel, Germany; 2 Department of Surgery, UKSH Campus Kiel, Kiel, Germany; 3 Technische Universität München, Klinikum rechts der Isar, II. Medizinische Klinik, Munich, Germany; 4 University Medical Center Göttingen, Department of General, Visceral and Pediatric Surgery, Göttingen, Germany; 5 Institute of Pathology, UKSH Campus Kiel, Kiel, Germany; 6 University Department for Gastroenterology, Klinikum Oldenburg AöR, European Medical School (EMS), Oldenburg, Germany; 7 Institute of Experimental Cancer Research, UKSH Campus Kiel, Kiel, Germany

**Keywords:** Pancreatic cancer, Pancreatic cancer

## Abstract

The emergence of resistance to systemic therapies in pancreatic ductal adenocarcinoma (PDAC) is still a major obstacle in clinical practice. Both, constitutive and inducible NF-κB activity are known as key players in this context. To identify differentially expressed and TRAIL resistance mediating NF-κB target genes, TRAIL sensitive and resistant PDAC cell lines were analyzed by transcriptome assays. In this context, A20 was identified as an NF-κB/RelA inducible target gene. Translational PDAC tissue analysis confirmed the correlation of elevated A20 protein expression with activated RelA expression in PDAC patients. In in vitro experiments, an elevated A20 expression is accompanied by a specific resistance toward TRAIL-mediated apoptosis but not to chemotherapeutic-induced cell death. This TRAIL resistance was attributed to A20´s E3-ligase activity-mediating Zink finger domain. Furthermore, the ubiquitin-binding scaffold protein p62 was identified as indispensable for the TRAIL-mediated apoptosis-inducing pathway affected by A20. The results of this study identify A20 as a possible therapeutic target to affect resistance to TRAIL-induced apoptosis in PDAC cells.

## Introduction

Though the knowledge of cancer biology has made significant progress during the last two decades, cancer remains the second leading cause of death worldwide [1, 2]. Among the malignant diseases, pancreatic ductal adenocarcinoma (PDAC) is the most lethal, exhibiting very limited therapeutic options. Less than 20% of the patients have an option of surgical tumor resection with a curative intention. For most cases with already advanced disease, conventional combination chemotherapy remains the standard of care. Here, side effects and response rates of only 20–30% demonstrate the need to improve therapeutic strategies. Besides conventional anti-cancer drugs and the established regimen (such as FOLFIRINOX), novel molecular targets and signaling pathways suitable for more efficient treatment concepts are intensively investigated. One of the pathways that is in the focus of interest relates to the anti-tumor potential of TNF-related apoptosis-inducing ligand (TRAIL), also known as Apo-2 ligand (Apo2L).

TRAIL can induce apoptosis via the extrinsic or intrinsic pathways, whereby the present apoptotic pathway is cell type-specific [3]. The binding of TRAIL to its surface-receptors TRAIL-R1 or TRAIL-R2 initiates the formation of a death-inducing signaling complex (DISC). Via their death effector domains (DED), members of the DISC facilitate the binding of caspase-8/10 and the subsequent initiation of the extrinsic apoptotic pathway. Moreover, caspase 8 can also activate the cleavage of the Bcl2 homology domain 3 interacting domain death agonist (BID) and activate the intrinsic apoptotic pathway [4]. Since TRAIL receptors are abundantly expressed in cancer cells, TRAIL-based strategies were considered promising in anti-tumor therapy. However, rising numbers of reports on pre-existing and acquired resistance of cancer cells against TRAIL-induced cell death accumulated. In this context, TRAIL resistance is often associated with abnormal TRAIL-receptor expression or increased quantities of decoy receptors competing for TRAIL binding. Furthermore, overexpression of apoptosis-regulating proteins like cellular FLICE-inhibitory protein (cFLIP), inhibitors of apoptosis proteins (IAPs), or BCL-2 family members are known factors influencing TRAIL sensitivity [5, 6]. Recently, post-translational modifications like phosphorylation, ubiquitination, or SUMOylation of DISC components were identified as an additional level affecting TRAIL sensitivity [7]. Thus, a better understanding of TRAIL-mediated apoptotic and anti-apoptotic signaling pathways is needed [3, 8] to exploit the full therapeutic potential of TRAIL agonistic therapy concepts.

In this context, the constitutive and inducible activity of the transcription factor NF-κB has been described as an important mediator of TRAIL resistance [9, 10]. Notably, special attention must be paid to the target gene-inducing NF-κB members RelA (p65), RelB, and c-Rel, all harboring the C-terminal transactivation domain (TAD) [11]. In many cancer cells, among them, PDAC cells, especially the induced NF-κB activity and the RelA subunit target genes confer TRAIL resistance [12]. Although the role of NF-κB in TRAIL resistance has been already demonstrated, the genes downstream of the NF-κB signaling pathway conferring resistance as well as the modalities of their induction and cellular action are still not well understood.

In the present study, we performed a genome-wide transcriptome screen for NF-κB target genes differentially expressed in TRAIL-resistant PDAC cells. Thereby A20 (also known as TNFAIP3) was identified as a strongly TRAIL-inducible RelA/NF-κB target gene that impairs TRAIL-activated apoptosis.

## Material and methods

### Cell culture

Handling of human pancreatic cancer cell lines Panc1 (RRID:CVCL_0480), PaTu8902 (RRID:CVCL_1845), and MiaPaca2 (RRID:CVCL_0428) were carried out as described [13]. Patu8988t (RRID:CVCL_1847) cells were cultured in DMEM high glucose (#P04-03500, PanBiotech, Aidenbach, Germany) supplemented with 2 mM L-glutamine (#P04-80100, PanBiotech), and 10% FCS (#F7524, Sigma Aldrich, Darmstadt, Germany). Unless otherwise stated, cells were treated with 100 ng/ml TRAIL (#ALX-201-073, Enzo Life Science, Lörrach, Germany). Cell lines were tested for Mycoplasma contamination by MycoAllert Kit (#LT07-418, Lonza, Basel, Switzerland). Human cell lines were authenticated by short tandem repeat (STR) profiling (Eurofins Genomics, Ebersberg, Germany).

### RNA preparation and realtime PCR

Total RNA from cell lines was isolated using the Monarch Total RNA Miniprep Kit (#T2010S, NEB, Frankfurt, Germany), and total RNA from snap-frozen and RNA-later-embedded PDAC patient tissue was isolated using the All Prep DNA/RNA kit (#80284, Qiagen, Hilden, Germany) according to the manufacturer’s instructions. Fluorescent dye-based realtime PCR was done by using Luna Universal qPCR Master Mix (#M3003, NEB). Primer sequences were listed in primer-list S1. Raw data were analyzed by CFX Maestro Software (Bio-Rad, Feldkirchen, Germany) and the ΔΔCt method was used.

### siRNA transfection

For siRNA transfection cells were seeded in 12 wells and transfected with Lipofectamine RNAiMax (#13778150, Invitrogen, Karlsruhe, Germany). In detail, 15 pMol control, RelA, A20, or p62 specific siRNA were mixed with 4 µl transfection reagent. For each gene target at least 2 different siRNAs were used: RelA: s11915, s11916; A20: s14259, s14261; p62: s16960, s16961 (all silencer select predesigned siRNAs; ThermoFischer Scientific, Waltham, MA, USA).

## Construction of A20 expression plasmids

See Supplementary Methods for details.

## Plasmid transfection

A total of 10^5^ cells were seeded in 12 wells. At a confluence of ~70% cells were transfected with 0.3 µg plasmid DNA by using Effectene transfection reagent (#301425, Qiagen, Hilden, Germany) according to the manufacturer´s instructions.

## CRISPR/Cas9 clones

See Supplementary Methods for details.

### Gel shift assays

See Supplementary Methods for details.

### Western blot

For western blot analysis, cells were washed and lysed in RIPA buffer (#9806, Cell Signaling Technologies, Boston, MA, USA) for 30 min on ice. Lysates were examined by immunoblotting as described [13] with the use of primary antibodies: A20 (1:1000, Abcam Cat# ab92324, RRID:AB_10561788), PARP (1:1000, Cell Signaling Technology Cat# 9542, RRID:AB_2160739), Caspase 8 (1:1000, Cell Signaling Technology Cat# 9746, RRID:AB_2275120), Caspase 3 (1:1000, Cell Signaling Technology Cat# 9665, RRID:AB_2069872), p62 (1:1000, Abcam Cat# ab91526, RRID:AB_2050336), β-actin (1:5000, Cell Signaling Technology Cat# 4970, RRID:AB_2223172), HSP90 (1:2500, Santa Cruz Biotechnology Cat# sc-13119, RRID:AB_675659), RelA (1:200, Cell Signaling Technology Cat# 8242, RRID:AB_10859369).

### ChIP assay

See Supplementary Methods for details.

### Genome-wide transcriptome profiling and cluster analysis, human PDAC expression datasets and Kaplan-Meier analysis

See Supplementary Methods for details.

### Immunohistochemistry

See Supplementary Methods for details.

### subG1 apoptosis assay (Nicoletti Assay)

For analyzing apoptosis with subG1 assay treated cells were fixed with 70% alcohol, stained with 20 µg/ml propidium iodide (#81845, Sigma) and 50 µg/ml RNAse (#556746, Merck) for 30 min. By FACS analysis (FACSVerse, BD, Heidelberg, Germany) cell fraction with a PI staining smaller than G1-fraction (subG1) were considered apoptotic cells.

### Caspase-3/-7 assay

Caspase-3/-7 activity (#G8090, Promega, Walldorf, Germany) was measured according to the manufacturer’s instructions and as described [13]. All assays were done in duplicates.

### Statistics

All experiments were conducted in biological quintuplicates unless otherwise stated. Data represent the mean ± standard deviation and were analyzed by two-sided *t*-test or by the Pearson correlation formula. *P*-values were calculated with Microsoft Excel (RRID:SCR_016137) and values less than <0.05 were considered statistically significant.

### Ethics statement

The research was approved by the ethics committee of the Medical Faculty of Kiel University (reference D 442/09) and written informed consent from patients for research was obtained before the investigation.

## Results

### A20 is an NF-κB/RelA target gene in resistant PDAC cell lines

**Fig. 1:**
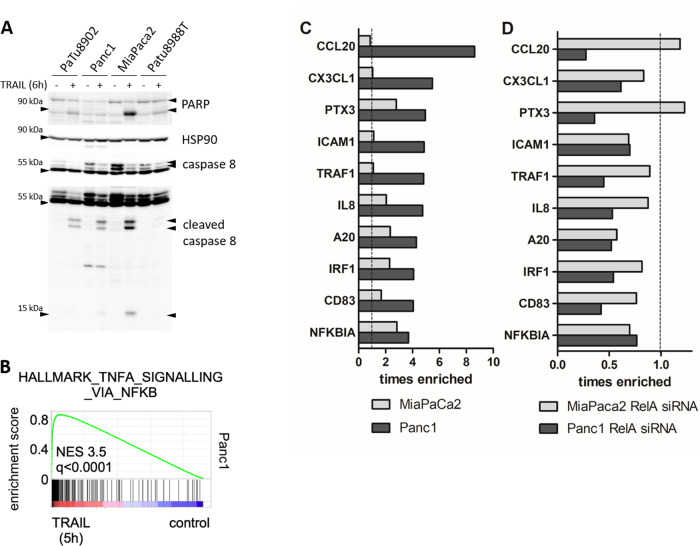
A20 is a TRAIL inducible target gene in TRAIL-resistant PDAC cells. **A** Cells were treated with TRAIL for 6 h and whole-cell lysates were analyzed by western blot with indicated antibodies. HSP90 was used as a loading control. *n* = 5. **B** GSEA of RNA-Seq expression data from human Panc1 cells treated with 10 ng/ml TRAIL for 5 h. Depicted is the HALLMARK signature TNFA SIGNALLING VIA NF-κB, including the q value. (C + D) PDAC cells were left untreated **C** or transfected with RelA-specific siRNA **D** for 48 h. Afterward, cells were treated as described in **B** and a genome-wide array was performed. Top 10 differentially TRAIL-induced genes of a genome-wide expression array in Panc1 and MiaPaca2 cells, ranked by fold change are depicted.

The inducible NF-κB activity is associated with resistance to chemotherapy and TRAIL-induced apoptosis in PDAC cells [14, 15]. Analyzing a panel of human PDAC cancer cell lines towards their responsiveness to TRAIL-induced apoptosis, MiaPaca2 cells were identified as TRAIL sensitive while Panc1, PaTu8902, and Patu8988t cells were considered resistant, as demonstrated by differential induction of caspase-8 activity and cleavage of the caspase substrate PARP (Fig. 1A).

To identify target genes and pathways upregulated after TRAIL treatment in resistant but not in sensitive PDAC cells, an unbiased genome-wide transcriptome profiling was performed. GSEA of this RNA-seq experiment confirmed a TRAIL-mediated activation of the TNF-SIGNALLING VIA NF-κB signature in resistant Panc1 cells (Fig. 1B). The comparison of TRAIL-induced gene expression in sensitive MiaPaca2 and resistant Panc1 cells identified among the top 10 differentially expressed genes (Fig.1C) chemokines (CCL20, CX3CL1), inflammation-associated genes (PTX3, IRF1), genes related to the NF-κB pathway (IL-8, NFKBIA), and genes, like TRAF1 and A20, that can assemble with death-receptor-associated complexes. Treating these PDAC cell lines with RelA-specific siRNA resulted in a reduced expression of these genes (Fig. 1D) while in sensitive MiaPaca2 cells only limited effects are detectable. The results indicate a participation of the 10 analyzed genes in NF-κB/RelA-mediated apoptosis resistance. Although no established apoptosis-associated gene was identified by the transcriptome profiling, we focused on A20 and its role in TRAIL resistance of PDAC cells, since its association with apoptosis resistance has been reported recently [16] besides its well-known NF-κB inhibitory function.

**Fig. 2:**
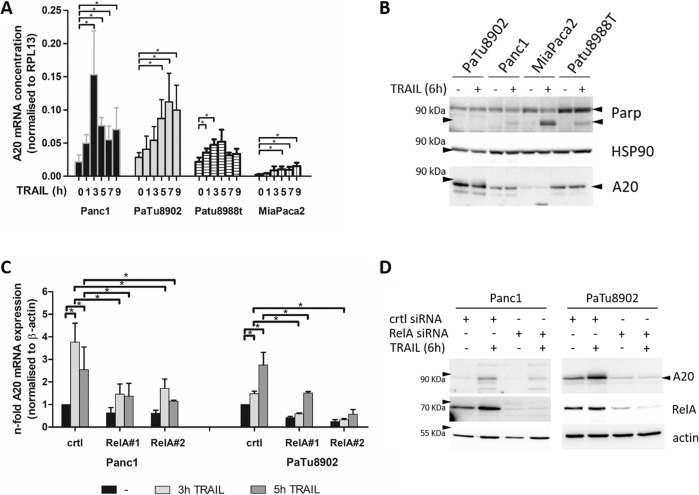
RelA-dependent A20 expression correlates with chemoresistance of PDAC cells. **A** Panc1, Patu8988t, PaTu8902, and MiaPaca2 cells were left untreated or treated with TRAIL for indicated periods. Isolated total RNA was submitted to reversed transcription and A20 mRNA was measured by real-time PCR. An external standard curve was applied. A20 mRNA expression was related to RPL13 gene expression. The mean values of 5 independent experiments performed in duplicates ± S.D. are depicted, *P-values <0.05. **B** Cells were treated with TRAIL for 6 h and whole-cell lysates were analyzed with indicated antibodies. HSP90 was used as a loading control, *n* = 5. (C + D) Resistant PDAC cells were treated with indicated siRNAs and left untreated or with TRAIL for 3 h or 5 h **C** or 6 h **D**. **C** Isolated total RNA was reversed transcribed and analyzed by real-time PCR for A20 mRNA expression and normalized to housekeeper gene expression. Depicted are the mean values of 5 independent experiments performed in duplicates ± SD *P-values < 0.05. **D** western blot analysis with total lysates was done with indicated antibodies and β-actin as a loading control, *n* = 5.

To verify the transcriptome analysis, A20 expression was analyzed by qPCR. PancTu1, Panc1, and Patu8988t cells exhibited a TRAIL-inducible A20 mRNA expression while sensitive MiaPaca2 cells exhibited no or only moderate A20 expression (Fig. 2A). Consistent with A20 mRNA expression, all tested TRAIL-resistant cell lines showed considerable A20 protein expression on a basal level as well as after TRAIL treatment. In contrast, MiaPaca2 cells reveal only little A20 protein expression under untreated or TRAIL-treated conditions. Analysis of PARP showed a significantly lower amount of cleavage in TRAIL-treated resistant PDAC cells, suggesting a negative correlation between A20 and TRAIL sensitivity (Fig. 2B). Furthermore, transfecting Panc1 and PaTu8902 cells with RelA-specific siRNA confirmed RelA dependency of the TRAIL-induced A20 expression on both mRNA and protein level (Fig. 2C, D).

### A20 and RelA expressions in pancreatic cancer tissues are elevated

**Fig. 3:**
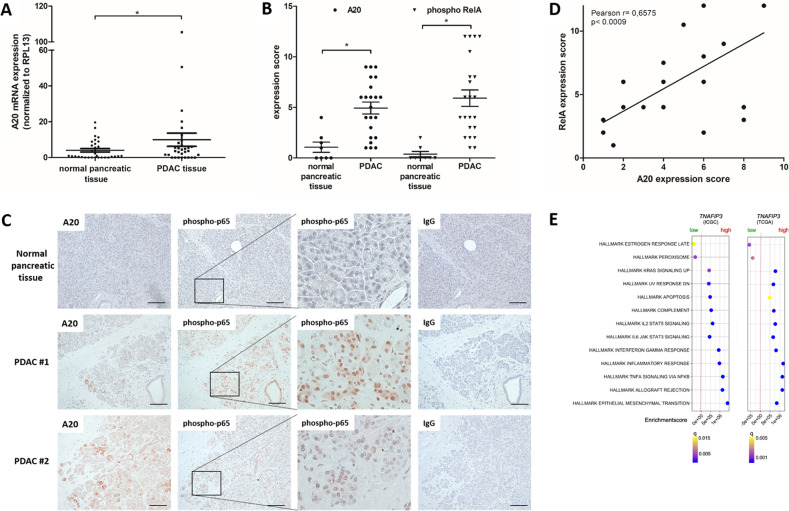
RelA and A20 expression are elevated in PDAC tissue. **A** Total RNA from 31 PDAC tissues and corresponding normal tissues were isolated, reversed transcribed, and analyzed by qPCR for A20 expression. For normalization RPL13 expression was analyzed. Displayed is the mRNA expression of tumor and normal tissue and the respective mean ± SD. *P-values <0.02. **B** Depicted is the expression score (ES = P*S) of A20 and phospho-RelA staining in 22 PDAC and 8 normal tissues ± SEM. *P-values < 0.05. **C** Representative images of immunohistochemical staining for A20, phospho-p65, and IgG-control (scale bar = 100 nm) of tissues from two PDACs and one normal pancreas are shown. **D** Depicted are the Pearson correlation coefficient and the linear regression of the A20 and phospho-RelA expression score (ES) of PDAC tissues (*n* = 22). *P*-value is indicated. **E** Results of GSEA hallmark analysis for A20 (TNFAIP3) high and low expressing PDAC of TCGA and ICGC Data.

In a translational approach, we analyzed the expression of A20 in PDAC tissues by qPCR analyses of mRNA samples and immunostainings. A total of 24 of 31 mRNA samples from PDAC specimens exhibited a higher A20 mRNA expression compared to the corresponding normal tissues (Fig. 3A).

Consecutive sections from 22 FFPE-tissues from PDAC patients and 8 normal pancreatic tissue were immunohistochemically stained with A20 and phospho-RelA-specific antibodies. While normal pancreatic tissues showed only weak staining for A20, a majority of PDAC tissues (15/22) exhibited a distinct expression of A20 in the cytoplasm of cancer cells and dysplastic ductal structures (Fig. 3B, C). Similar results can be seen for phospho-RelA-specific staining. The majority of PDAC sections (16/22) showed considerable nuclear phospho-RelA staining while the nucleus of normal pancreatic tissues showed only slight staining (Fig. 3B, C), suggesting a more pronounced transcriptional activity of NF-κB in PDAC tissues. The determined expression scores were used for Pearson correlation analysis and showed a positive correlation between A20 and phospho-RelA expression in PDAC tissues (Fig. 3D). To corroborate these findings, we further analyzed PDAC mRNA expression datasets. We compared PDAC with high A20 mRNA expression to PDAC with low expression by gene set enrichment analysis and observed activation of inflammation-associated hallmarks in cancers with high A20 expression (Fig. 3E).

### TRAIL stimulates the binding of RelA to the A20 promoter

**Fig. 4:**
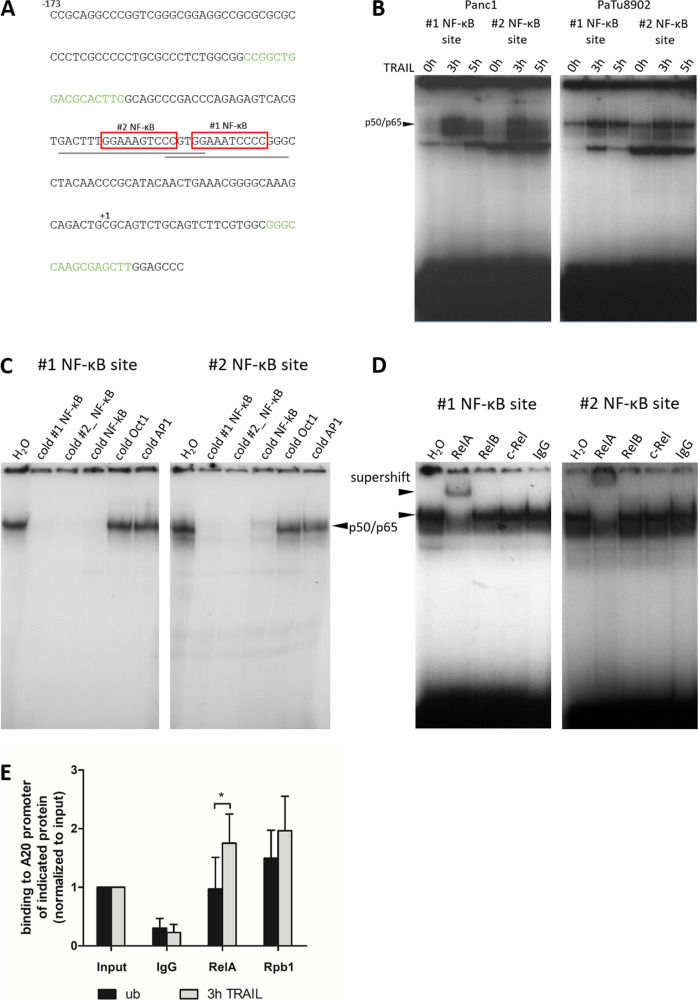
NF-κB/RelA binds to the A20 promoter. **A** Sequence of the A20 5´flanking region containing two κB elements (highlighted in boxes; #1 NF-κB site: −45 to −54 (GGAATCCCC) and #2 NF-κB site: −57 to −66 (GGAAAGTCCC)). Underlined are the EMSA oligos in grey and the ChIP primers are marked in green. **B** Panc1 or PaTu8902 cells were treated with TRAIL for indicated times. Nuclear extracts were analyzed by EMSA assays with oligonucleotides spanning one putative κB binding site, *n* = 2. **C** For competitive EMSA assays, TRAIL-treated PaTu8902 nuclear extracts were incubated in addition to the indicated ^32^P-labeled NF-κB probes with non-labeled #1/#2 NF-κB oligonucleotides, a consensus NF-κB probe, or two unrelated probes (Oct1 and AP1), *n* = 2. **D** For supershift experiments with Panc1 nuclear extracts indicated antibodies were used, *n* = 2. **E** Panc1 cells were treated with TRAIL for 3 h or left untreated and lysates were examined by ChIP assay using IgG, RelA, or Rpb1 antibody for precipitation. Primers spanning both κB sites and results were normalized to input. Mean ± SD of 4 independent experiments is shown. *p < 0.02.

Various publications describe the activation of the A20 promoter by NF-κB under the control of different stimuli [17–19]. In this context, sequence analysis identified two putative NF-κB binding sites within the A20 promoter (Fig. 4A). Electrophoresis mobility shift assay (EMSA) experiments with binding site-specific radiolabeled oligonucleotides TRAIL treatment for 3 h or 5 h induced the binding of the p50/p65 heterodimer to both putative sites in Panc1 or PaTu8902 cells after treatment of 3 h or 5 h (Fig. 4B). Competitive EMSA experiments, using both A20 oligonucleotides as well as a commercially available NK-κB consensus probe, displaced the binding of the NF-κB heterodimer to both radiolabeled oligonucleotides, while the addition of unrelated oligonucleotides had no effect to the binding (Fig. 4C). To confirm RelA as a major part of the heterodimeric complex as well as to exclude the NF-κB subunits RelB and c-Rel as interaction partners with p50 in the heterodimer, supershift experiments were performed. While the addition of RelA-specific antibodies resulted in the binding of the oligonucleotide-NF-κB complex and a subsequent supershift, specific antibodies to RelB or c-Rel had no effect (Fig. 4D). This indicates that the canonical RelA/p50 NF-κB complex activates the A20 gene in response to TRAIL stimulation. Consistently, quantitative chromatin immunoprecipitation assays (ChIP Assays) confirmed the binding of RelA to the NF-κB binding sites after TRAIL stimulation. In addition, the transcriptional activation of this A20 promoter region after TRAIL treatment was verified by binding of RNA polymerase II (RpbI, largest subunit of RNA polymerase II) (Fig. 4E). Due to the proximity of the two analyzed binding sites (see Fig. 4A) a clear distinction of RelA binding to a single or both NF-κB binding sites is not possible by ChIP assay.

### A20 mediates resistance towards TRAIL but not to etoposide or gemcitabine in PDAC cells

**Fig. 5:**
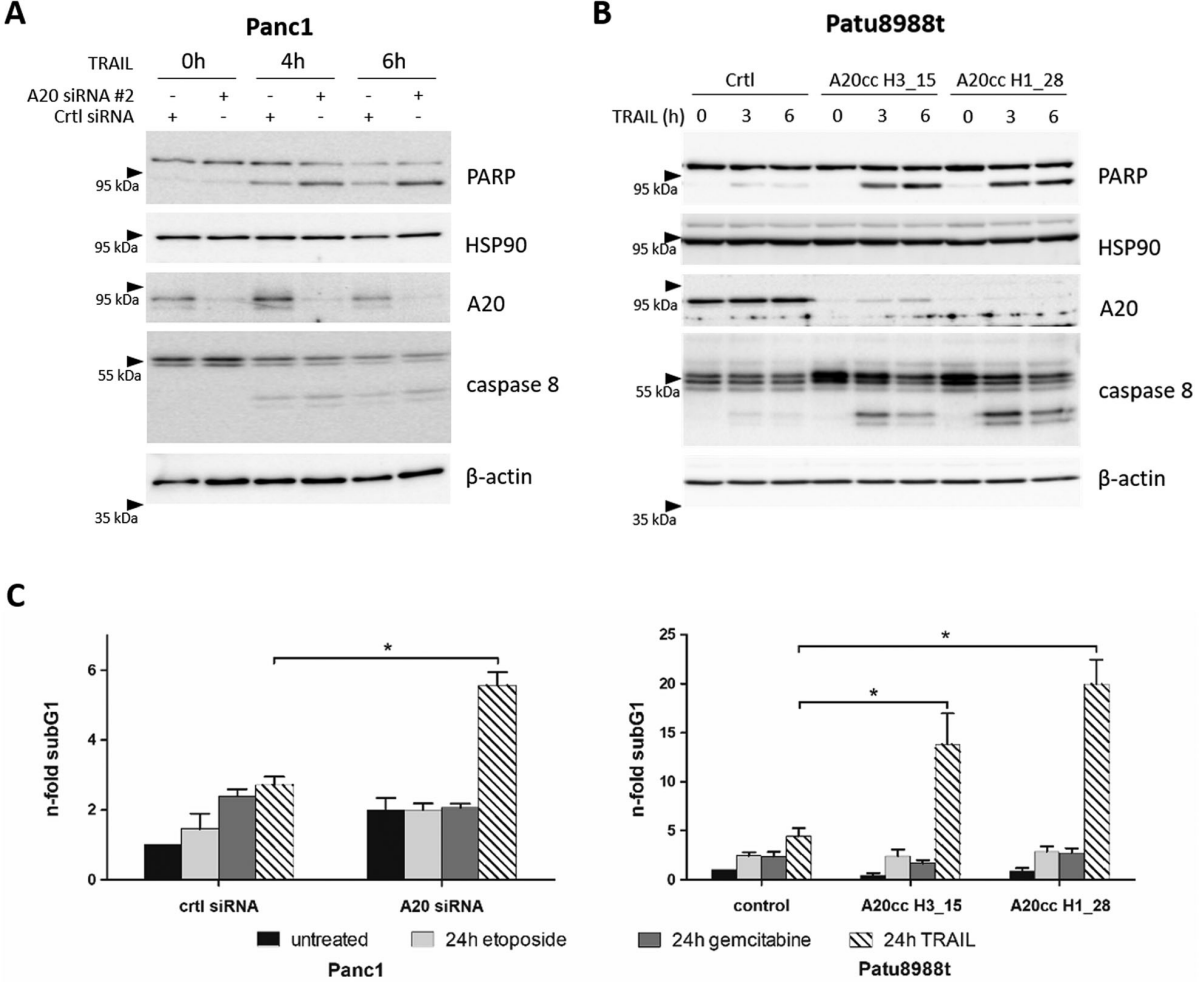
A20 mediates chemoresistance towards TRAIL in PDAC cells. SiRNA-treated Panc1 cells (left panel) or Patu A20cc (right panel) were treated with therapeutic drugs (20 µg/ml etoposide, 10 µg/ml gemcitabine, or 100 ng/ml TRAIL) for indicated periods. **A**, **B** whole-cell lysates were conducted to western blot analysis with indicated antibodies and HSP90 and β-actin as loading controls, *n* = 5. **C** Panc1 cells (left panel) and Patu8988t (right panel) were analyzed for apoptotic cell death by subG1 assay. Shown are results from 5 independent experiments, *p < 0.05, *n* = 5.

To analyze the function of A20 mediating resistance towards TRAIL-induced apoptosis we made use of specific siRNAs knocking down the A20 expression in Panc1 cells as well as a CRISPR/Cas9 mediated knock-down of A20 in Patu8988t (Patu A20cc) cells were established. Off-target activity of Cas9 depends amongst others on sgRNA sequences [20]. To minimize this effect two different A20-specific sgRNA (H1 and H3) were used and PatuA20cc H1 and PatuA20cc H3 cell lines were generated. Both approaches showed a significant reduction of A20 at the protein level in Panc1 and Patu8988t cells (Fig. 5A, B). The knock-down of A20 expression was associated with the sensitization of both cell lines to TRAIL treatment as shown by caspase 8 and PARP cleavage (Fig. 5A, B).

Analyzing the impact of A20 on the effectiveness of different chemotherapeutic agents, A20 siRNA-treated Panc1 and Patu A20cc were treated with gemcitabine, etoposide, or TRAIL. Consistently, the control-siRNA or CRISPR/Cas9 control-treated cells show a resistant phenotype towards all tested chemotherapeutic drugs and TRAIL. Notably, the reduction of A20 by siRNA or via CRISPR/Cas9-mediated knockdown did not affect the sensitivity towards gemcitabine or etoposide in the two PDAC cell lines (Fig. 5C) whereas a significant sensitization towards TRAIL-mediated apoptosis was observed (Fig. 5C). These unexpected results point out a highly specific function of A20 in mediating resistance to death receptor-associated apoptosis but not to chemotherapeutic drugs.

### A20´s ubiquitin E3-ligase Znf4 domain mediates chemoresistance towards TRAIL

**Fig. 6:**
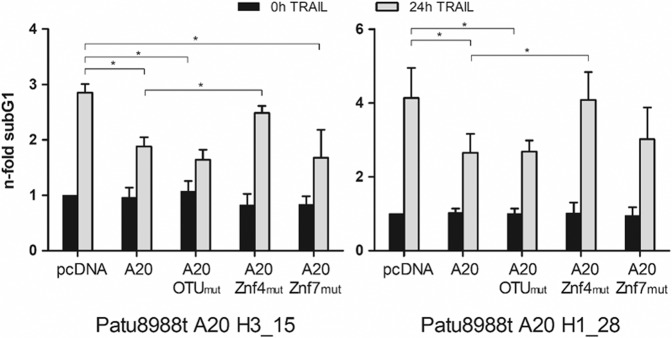
Znf4 domain of A20 accountable for chemoresistance mediation. Patu A20cc cells were transfected with indicated A20 expression plasmids, treated with TRAIL for 24 h, and analyzed by subG1 assay. Plotted are mean ± S.D. from 5 independent experiments.

A20 is a ubiquitin (Ub) modifying enzyme acting on one hand as a deubiquitinating enzyme and on the other hand as an E3 ubiquitin ligase. While the deubiquitinating function is conferred by the ovarian tumor domain (OTU), the fourth zinc finger domain (Znf4) has an Ub E3-ligase activity. Besides these enzymatic functions, A20 can also exhibit a non-enzymatic function by binding to ubiquitin chains through its Znf4 or Znf7 domain, thereby modulating cellular signaling [21]. Since all of these domains have been implicated in pro- and anti-apoptotic signaling pathways, the individual impact of one particular domain in mediating TRAIL resistance in PDAC cells was analyzed. Patu A20cc cells were transfected with a plasmid for wild-type A20 or plasmids expressing A20 with mutations in the OTU-domain, the Znf4-domain, or Znf7-domain, respectively. While the transfection with wild-type A20, as well as with the A20-OTU-mutant or A20-Znf7-mutant restored TRAIL resistance in PatuA20cc cells, the expression of A20-Znf4 mutated protein did not affect TRAIL-induced apoptosis (Fig. 6). This observation points to a central role of the Znf4-domain in TRAIL resistance of PDAC cells.

### A20-mediated TRAIL resistance is associated with a p62-dependent caspase 8 activation and apoptosis induction of PDAC cells

**Fig. 7:**
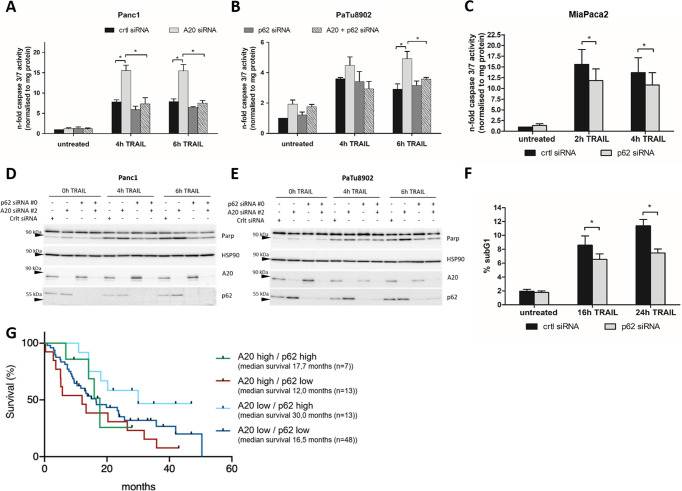
p62 is needed for TRAIL-mediated apoptosis induction. **A**, **C** Panc1 and **B**, **D** PaTu8902 cells were transfected with control, A20, p62, or A20 + p62 siRNA and treated with TRAIL for 0 h, 4 h, or 6 h. **A**, **B** caspase 3/7-assay was performed in duplicates and normalized to mg protein. Depicted are the mean ± SD from 5 independent experiments. **C**, **D** whole-cell lysates were analyzed by western blot experiments with indicated antibodies and HSP90 as a loading control, *n* = 5. **E**, **F** MiaPaca2 cells were transfected with control or p62 siRNA and subsequently treated with TRAIL for indicated periods. **E** caspase 3/7-activity was measured and normalized to mg protein. **F** cells were fixed with ethanol and analyzed by subG1 assay. Depicted are means ± SD from 5 independent experiments. **G** Survival of patients with high (>75^th^ percentile) and low (<25^th^ percentile) A20 mRNA expression were subdivided by their p62 mRNA expression (high (>75^th^ percentile), low (<25^th^ percentile)). Clinical data based on the curated ICGC data set (*n* = 81)).

In head & neck squamous cell carcinoma cells, the autophagic cargo adapter p62/SQSTM1 is augmenting the activation and full processing of caspase 8 by capturing ubiquitinated caspase 8 into aggresome-like structures, necessary for subsequent apoptosis induction upon radiation treatment [22]. These findings raised the question if p62 is part of the apoptosis-inducing signaling pathway which is affected by A20 in TRAIL-resistant PDAC cells. As already shown (Fig. 5C), siRNA-mediated silencing of A20 sensitized resistant PDAC cells to TRAIL-induced apoptosis. In contrast, siRNA-mediated silencing of p62 expression did not affect TRAIL sensitivity. However, simultaneous reduction of A20 and p62 protein expression prevented the sensitizing effect of A20 downregulation towards TRAIL-induced apoptosis (Fig. 7A, B). At the protein level, the A20 siRNA-mediated sensitizing effect to TRAIL-induced apoptosis was repealed by simultaneous siRNA-mediated p62 reduction, too (Fig. 7C, D).

In line with these results, TRAIL-sensitive and low quantities of A20 expressing MiaPaca2 cells gain resistance to TRAIL-induced cell death due to reduced p62 expression (Fig. 7E, F). To further substantiate these findings, ICGC clinical data sets were analyzed. The Kaplan-Meier curves of PDAC patients with high and low A20 mRNA levels were subdivided by their p62 mRNA expression. In A20 low and p62 low expressing tumors median survival time is nearly bisected compared to A20 low/ p62 high mRNA expressing tumors (A20 low/p62 high: 30 months vs. A20 low/p62 low: 16,5 months) (Fig. 7G). These results point out that the ubiquitin-binding scaffold protein p62 is part of the TRAIL-mediated apoptosis signaling pathway in PDAC cell lines and can be affected by A20.

## Discussion

PDAC is estimated to become the second leading cause of cancer-associated death by 2030 [23]. At the same time, treatment options for PDAC patients are still limited and an understanding of occurring resistances is needed. In this context, NF-κB activity was identified as a key player responsible for acquired resistance against gemcitabine or topoisomerase inhibitors in PDAC [10, 24]. Likewise, death receptor-mediated apoptosis in PDAC cells is also reduced by an inducible NF-κB activity, thereby conferring resistance to death ligands like TNF-α, TRAIL, or FASL [15]. In view of the huge range of stimuli that activate NF-κB as well as the plethora of its target genes [25, 26], the inhibition of NF-kB is rather a delicate issue. Therefore, the knowledge of resistance-mediating NF-κB downstream signaling pathways may offer an option to develop or adopt clinical applications. In the present study, we identified A20 as a highly expressed and TRAIL-resistance mediating NF-κB/RelA target gene in PDAC cells.

Expression analysis of TRAIL-sensitive and resistant PDAC cell lines identified A20 as a differentially expressed TRAIL inducible NF-κB/RelA target gene. Initially, A20 was characterized as a primary response gene to TNF treatment in endothelial cells [27] and is known as a negative feedback regulator of NF-κB activation [28]. However, its ability to modify ubiquitin-dependent signaling pathways has made A20 a versatile regulator acting in a context-dependent manner [21, 29–31].

A20 expression is controlled by transcriptional, posttranscriptional, and posttranslational mechanisms in several cells, and a transient and rapid A20 expression is induced by NF-κB signaling [16]. Consistently, immunohistochemically analyzed PDAC tissues showed elevated nuclear staining of phospho-RelA correlating with elevated A20 staining. Furthermore, the TRAIL-induced binding of a p65/p50 heterodimer to two NF-κB response elements within the A20 promoter and a subsequent protein expression identified the participation of the inducible NF-κB activity in A20 expression in TRAIL-resistant PDAC cell lines.

Regarding chemoresistance, several studies have shown different effects of A20 in a cancer-cell type anti-cancer-drug specific response. A20 overexpressing MCF-7 breast cancer cells showed in comparison to wild-type MCF-7 cells an enhanced resistance towards the mitotic inhibitor taxol, while the cells showed no difference in response to gemcitabine-induced apoptosis induction [32]. In contrast to this, a knockdown of A20 promoted chemoresistance to gemcitabine in SW1990 PDAC cells [33]. In another study, a reduced A20 expression in several cancer cell lines affected DNA repair mechanisms and sensitized cells towards etoposide or irradiation-induced DNA damage [31]. For the PDAC cells analyzed in the present study, we ruled out an effect of A20 on the resistance to gemcitabine or etoposide. However, a reduced A20 expression sensitized the PDAC cells towards TRAIL-mediated apoptosis suggesting a specific role of A20 in death receptor-mediated apoptosis.

A20 acts as a ubiquitin editing enzyme containing both a deubiquitinating (OUT) and an E3-ligase (Znf4) domain. One of the substrates of A20-mediated ubiquitin editing is RIP1. By replacing K63-linked polyubiquitin with K48-linked polyubiquitin A20 favors proteasomal degradation of RIP1 [34]. Consequently, A20 leads to an inhibition of caspase 8 activation and subsequently to resistance to TRAIL-induced apoptosis in glioblastoma or hepatocellular carcinoma cells [35, 36]. A20 can probably modulate cellular signaling also in a non-enzymatic way by binding to ubiquitin chains via the Znf domains, acting as a scaffold/ubiquitin protective protein. In this process, A20-Znf4 exhibits a higher affinity to K63-linked polyubiquitin chains while A20-Znf7 preferentially binds to M1-linked polyubiquitin chains [37]. LUBAC, the only known E3-ligase to form linear polyubiquitin chains, is required for the recruitment of A20 and CYLD to the TRAIL complex I and II and subsequently affects the activity of apoptosis- and necroptosis mediating signaling complexes [38]. To test whether the OTU or Znf domains of A20 mediate TRAIL resistance in PDAC cells, Patu A20cc cells were transfected with wild-type A20 (A20wt) or inactive A20 mutants (Znf7mut, Znf4mut, OTUmut). Except for Znf4mut transfected cells, all A20 constructs conferred resistance towards TRAIL-induced cell death, suggesting a particular dependency of TRAIL resistance on the Znf4 domain of A20.

TRAIL treatment of the resistant PDAC cells showed an A20-mediated reduced procaspase-8 activation. In gastric epithelial cells, full caspase-8 activity is associated with its K63-linked polyubiquitination, affected by A20 [39]. Moreover, the multifunctional scaffold protein p62/SQSTM1 is known to facilitate full activation of ubiquitinated procaspase-8 in TRAIL-stimulated cells by trapping ubiquitinated procaspase-8 in p62-dependent foci [40]. Both, in resistant A20 siRNA-treated PDAC and in sensitive, little A20 expressing MiaPaca2 cells, p62 is a prerequisite for TRAIL-induced apoptosis induction. This points to the participation of p62 in TRAIL-mediated cell death and provides the essential link to the modulating effect of A20 on TRAIL sensitivity.

In conclusion, the presented data identify A20 as an NF-κB/RelA-induced TRAIL-resistance mediating protein in PDAC cells. Targeting A20´s E3-ligase activity may be a starting point for therapy concepts evading TRAIL resistance mechanisms in PDAC while retaining p62 scaffolding activity must be ensured.

## Supplementary information

Supplementary Methods

Checklist

Original Data File

## Data Availability

All datasets generated and analysed during this study are included in this published article and its Supplementary Information files. Additional data are available from the corresponding author on reasonable request.
