# Supplementary material for: NF-κB/RelA controlled A20 limits TRAIL-induced apoptosis in pancreatic cancer
Source: Cell Death Dis. 2023 Jan 3;14(1):3. doi: 10.1038/s41419-022-05535-9 (PMC9810737; doi:10.1038/s41419-022-05535-9)
Supplement: Supplementary file 1 — Supplementary Methods [file 41419_2022_5535_MOESM1_ESM.pdf]

Supplementary Methods

Gel shift assays

For Gel shift assays γ^{32}-P-labeled oligonucleotides (#1 NF-κB: CCGTGGAAATCCCCGGG; #2 NF-κB: GACTTTGGAAAGTCCCGTGG) harboring the underlined NF-κB recognition sites according to TFM-explorer (https://bioinfo.lifl.fr/tfm-explorer/tfm-explorer.php) and [17,18] were used. In detail, 1x 10^6 TRAIL-treated Panc1 or PaTu8902 cells were lysed in 1,5 ml EMSA buffer 1 (10 mM HEPES, pH7,9; 10 mM KCl; 0,2 mM EDTA; 1 mM DTT; 0,6% Triton X-100 (v/v); 1 mM PMSF; 10 µg/ml Aprotinin (#A126.1, Carl Roth, Karlsruhe, Germany); 1% phosphatase inhibitor cocktail II and III (v/v) (#P5726, #P0044, Sigma), centrifuged at 13.000 x g for 1 min at 4°C and the supernatant was discarded. After washing the pellet twice with 1 ml EMSA I buffer (without TX-100), the pellet was suspended in 100 µl EMSA Buffer II (20 mM HEPES, pH7,9; 0,4 M NaCl; 0,2 mM EDTA; 1 mM DTT; 1 mM PMSF; 10 µg/ml Aprotinin; 1% phosphatase inhibitor cocktail II and III), gently shaking for 20 min and centrifuged for 15 min at 13000 x g, both at 4°C. The nuclear protein-containing supernatant was collected.
For labeling the oligonucleotides, annealed oligonucleotides were labeled with γ^{32}-P by incubating 13 µl aqua dest, 2 µl oligonucleotide (2 pMol/µl), 2 µl T4-Kinase buffer, 1 µl T4-Kinase (#M4101, Promega, Walldorf, Germany), and 2 µl γ^{32}-P-ATP (#P-RP-1-L, Hartmann Analytic, Braunschweig, Germany) for 30 min at 37°C. Afterward, the labeled probe was separated by purification with Nick-Columns (Sephadex G50, #17-0855-02, Sigma). The volume of the labeled probe was reduced by vacuum centrifugation to 40 µl.

For EMSA experiments, 5 µg of nuclear proteins were incubated with 2 µl 5xbinding buffer (# E3581, Promega), 2 µl of the labeled probe, and adjusted to a final volume of 11 µl. For supershift experiments, 2 µl of indicated antibodies (RelA Santa Cruz Biotechnology Cat# sc-372X, RRID:AB_632037, RelB Santa Cruz Biotechnology Cat# sc-226X, RRID:AB_632341, c-Rel Santa Cruz Biotechnology Cat# sc-71X, RRID:AB_2253705), for competitive EMSA experiments 2 µl of the unlabelled probe (NF-κB consensus: #329B, Oct1 consensus: #E324B, AP1 consensus: #E320B, all Promega) were added and incubated at room temperature for 30 min. Afterward, samples were separated in a cooled 5 % native PAA gel (5 ml 40% PAA, 1,25 ml glycerol (v/v), 2 ml 10x TBE (#B52, ThermoFischer Scientific), 300 µl 10% ammonium peroxodisulfate (w/v), 30 µl tetramethylethylenediamine (w/v), adjusted to a volume of 40 ml) at 12 mA for 90 min. Gels were dried for 120 min at 80°C and submitted to radiography.

Construction of A20 expression plasmids:

Human cDNA was submitted to PCR using A20-Ex-F/R primers spanning the 2773 bp A20 coding sequence. The product was cloned by TA-cloning into pCR2.1 vector (#K202020, Invitrogen) and subsequently by using KpnI (#R0142) and XhoI (#R0146, both NEB) restriction enzymes into expression plasmid pcDNA3.1-Zeo (#V86020, Invitrogen). Mutations of OTU (C103A), Znf4 (C624A, C627A), and Znf7 (C799A, C782A) were inserted by using the Q5 site-directed mutagenesis kit (#E0554, NEB) according to the manufacturer's instructions. Primers used for mutations are listed in primer-list S1. All plasmids were verified by sequencing.

CRISPR/Cas9 Clones:

CRISPR/Cas9 mediated knockdown cells were established as previously described [14] using A20-specific sgRNAs A20-H1 (5'-CACGCAACTTTAAATTCCGC-3') and A20-H3 (5'-TTGCTCAAATACAAAGCCTG-3'). Oligonucleotides were synthesized with BsmbI restriction-site compatible overhangs, annealed, and inserted into the lentiCRISPR v2 puro (RRID:Addgene_98290) vector by Golden Gate Assembly. Sequences were confirmed by Sanger Sequencing.

For lentivirus production, HEK293FT cells (RRID:CVCL_6911) were transfected with the lentiCRISPR v2 vector harboring the sgRNA and the packaging plasmids psPAX2 (RRID:Addgene_12260) and pMD2.G (RRID:Addgene_12259). Media was changed 24 hours post-transfection to DMEM with 30% FBS. The supernatant, containing the virus, was collected after 48 hours and filtered through a 0.45 µm filter. Patu8988t cells were transduced with lentiviral supernatant containing 8 µg/ml Polybrene and selected with 3 µg/ml Puromycin 48 hours post-transduction. After 3 days of selection, single-cell clones were seeded out by limited dilution. Successful knockdown of A20 was determined by Western Blotting.

ChIP assay

ChIP assays were performed by using SimpleChIP Enzymatic Chromatin IP-Kit (#9003, Cell-Signaling Technologies) following the manufacturer´s instructions. DNA from Panc1 cells was immunoprecipitated with either 2 µg of anti-RelA (Cell Signaling Technology Cat# 8242, RRID:AB_10859369), anti-Rpb1 (Cell Signaling Technology Cat# 2629, RRID:AB_2167468), or rabbit IgG (Cell Signaling Technology Cat# 2729, RRID:AB_1031062). Primer sequences are listed in primer-list S1.

Immunohistochemistry

All tissues were from patients with PDAC who had undergone surgery (Whipple procedure) for PDAC resection. Conservation and histopathological diagnosis were performed at the Institute of Pathology, UKSH Campus Kiel. Only tissues pathologically staged T3N1M0 were included in the study. Formalin-fixed and paraffin-embedded (FFPE) tissues from PDAC and normal pancreatic tissue were immunostained with anti-phospho-RelA (1:500, Abcam Cat# ab86299, RRID:AB_1925243), anti-A20 (1:100, (Abcam Cat# ab92324, RRID:AB_10561788) or with isotype-matched control antibody (Cell Signaling Technology Cat# 2729, RRID:AB_1031062). In detail, slides were deparaffinized and rehydrated. Antigen retrieval was done with Citrate buffer (RelA; 1,8mM Citric acid, 8,2 mM Sodium citrate) or TRIS-EDTA (A20; 10mM Tris, 1 mM EDTA, 0,005 % Tween20) for 20 min at 95°C. After blocking with 4% BSA (#11926, Serva, Heidelberg, Germany) in 0,3% Triton X-100 (#1610407, BioRad), in TBS (25mM Tris, 2 mM KCl, 150mM NaCl, pH 7,4) for 30 min at room temperature (RT)the slides were incubated with a specific primary antibody at 4°C overnight in a moist chamber. For detection, sections were incubated for 30 min at RT with SignalStain® Boost IHC Detection Reagents (#8114, Cell Signaling) and substrate reaction was performed with AEC Substrate (#K3461 DAKO, Hamburg, Germany) for 20 min. Sections were stained with Mayer´s Haemalaun (#109249 Merck, Darmstadt, Germany) for 1 min and covered with Kaiser´s glycerine gelatine (#3G-031, Waldeck, Muenster, Germany). Images were captured by using the microscope Axio Imager M.2 with Axiocam 305 color and analyzed using the AxioVision Software (Carl Zeiss,

Jena, Germany). For evaluation, sections were assessed on the intensity of staining (0: no staining; +1: weak; +2 medium, +3 strong) and the average percentage of staining in 5 areas (0:<5%, 1: 5-24%; 2: 25-49%, 3: 50-75%; 4: >75 %). The expression score (ES) was calculated by the equation ES = P*S.

Genome-wide transcriptome profiling and cluster analysis, human PDAC expression datasets, and Kaplan-Meier analysis

Genome-wide transcriptome profiling was performed as described in [18]. All transcriptome data were processed according to MIAME standards and made publicly available by submitting to NCBI GEO (series accession: GSE87287; http://www.ncbi.nlm.nih.gov/geo/query/acc.cgi?acc=GSE87287). For the gene set enrichment analysis (GSEA) of this dataset, data were analyzed with Geo2R (https://www.ncbi.nlm.nih.gov/geo/info/geo2r.html). The log FC was used as a rank to run a pre-ranked GSEA via the GSEA_4.1.0 web app with the HALLMARK gene sets (h.all.v.7.5) [19,20].

The PAAD dataset of the TCGA (RNA-seq V2) was downloaded via cBioPortal (http://www.cbioportal.org/) (12/2018) and curated as described [21,22]. Normalized human PDAC RNA-seq data (ICGC) were directly retrieved from the supplementary information of Bailey et al. [23] and curated as described [24]. Gene set enrichment analysis (GSEA) was conducted via the Gene Trail3 web tool (http://genetrail.bioinf.uni-sb.de/) [25]. In both datasets, PDACs with *TNFAIP3* (A20) mRNA expression >75th percentile were compared to cancers with *TNFAIP3* (A20) mRNA expression <75th percentile. HALLMARK gene sets (MSigDB: H) enriched or depleted with an adj. p. value <0.05 in both datasets were visualized. Clinical data of the curated ICGC data set (n=81) [24] were retrieved via the supplemental tables of Bailey et al. [23]. Survival was compared according to the following groups: (B) *TNFAIP3* (A20) high / *SQSTM1 (p62)* high: both mRNAs >75th percentile; *TNFAIP3* (A20) high / *SQSTM1 (p62)* low: *TNFAIP3* (A20) mRNA >75th percentile, *SQSTM1 (p62)* mRNA <75th percentile; *TNFAIP3* (A20) low / *SQSTM1 (p62)* high: *TNFAIP3* (A20) mRNA <75th percentile, *SQSTM1 (p62)* mRNA >75th percentile; *TNFAIP3* (A20) high / *SQSTM1 (p62)* high: both mRNAs <75th percentile. Groups were investigated by a Kaplan-Meier analysis conducted with PRISM 8. The p values were calculated by a log-rank test.

Primer-list S1

Realtime Primer Sequences:

A20-F: 5´- GCGTTCAGGACACAGACTTG -3´

A20-R: 5´- TGTCCCATTCATCATTCCAGTT -3´

p62-F: 5´-TGTGTAGCGTCTGCGAGGGAAA-3´

p62-R: 5´- AGTGTCCGTGTTTCACCTTCCG-3´

RelA-F: 5´- ACCGCTGCATCCACAGTTTC-3´

RelA-R: 5´-GGGGTTGTTGTTGGTCTGGA-3´

RPL13-F:5´-CCTGGAGGAGAAGAGGAAAGAGA-3´

RPL13-R: 5´-TTGAGGACCTCTGTGTATTTGTCAA-3

β-actin-F: 5´-CTCTTCCAGCCTTCCTTCCT-3´

β-actin-R: 5´-AGCACTGTGTTGGCGTACAG-3´

Expression Plasmid Primer Sequences:

A20-Ex-F: 5´-ATGGCTGAACAAGTCCTTCCT-3´

A20-Ex-R: 5´- TTAGCCATACATCTGCTTGAACTG-3´

OTU-F: 5´-GAACGGTGACGGCAAT**gcc**CTCATGCATGCCAC-3´

OTU-R: 5´-GTGGCATGCATGAG**ggc**ATTGCCGTCACCGTTC-3´

Znf4-F: 5´-CTG**gcT**TTCATCGAGTACAGAGAAAAC-3´

Znf4-R: 5´-TGT**Agc**AAAGCCCTTGTTTTCTGG-3´

Znf7-F: 5´-GAA**gcC**TTTCAGTTCAAGCAGATGTATGG-3´

Znf7-R: 5´-GTTG**gcG**TAGCCGTTGCACTTGGC-3´

Primers for Chip Assay

A20-Chip-F: 5´-CCGGCTGGACGCACTTC-3´

A20-Chip-R: 5´-AAGCTCGCTTGGCCCG-3´

CRISPR/Cas9 plasmid synthesis primer:

A20-H1-F: CACCGCACGCAACTTTAAATTCCGC

A20-H1-R: AAACGCGGAATTTAAAGTTGCGTG

A20-H3-F: CACCGTTGCTCAAATACAAAGCCTG

A20-H3-R: AAACCAGGCTTTGTATTTGAGCAAC

All Oligonucleotides were purchased from Eurofins Genomics, Ebersberg, Germany.
