# Supplementary material for: NF-κB/RelA controlled A20 limits TRAIL-induced apoptosis in pancreatic cancer
Source: Cell Death Dis. 2023 Jan 3;14(1):3. doi: 10.1038/s41419-022-05535-9 (PMC9810737; doi:10.1038/s41419-022-05535-9)

Abbildung 1A

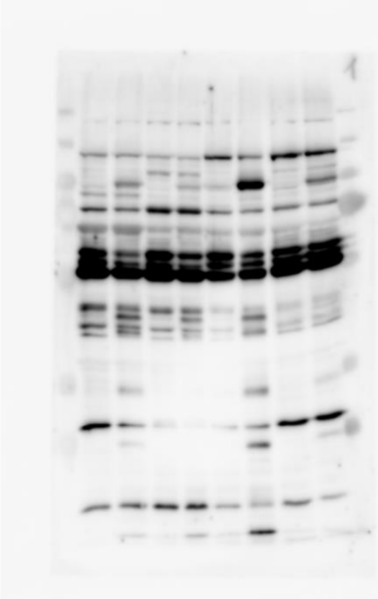

Parp

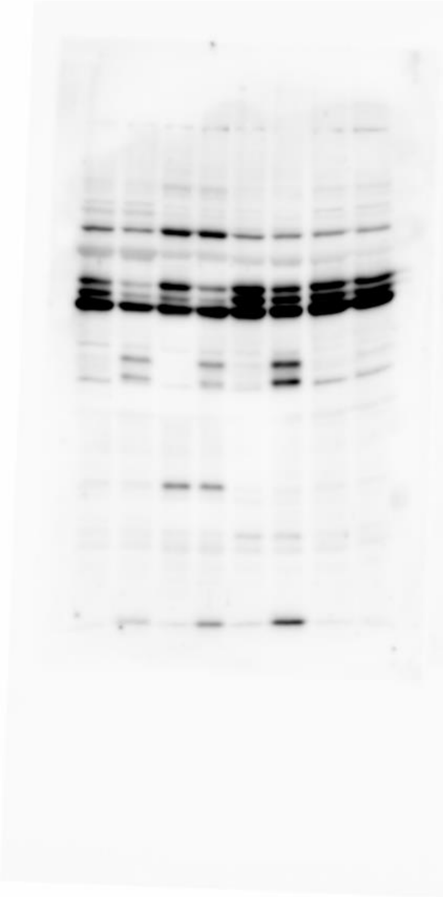

Caspase 8

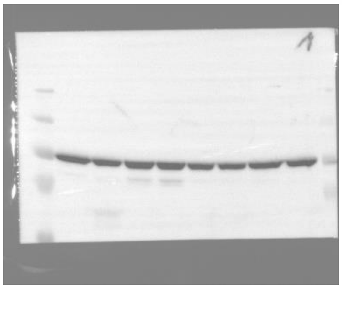

HSP90

Abbildung 2B

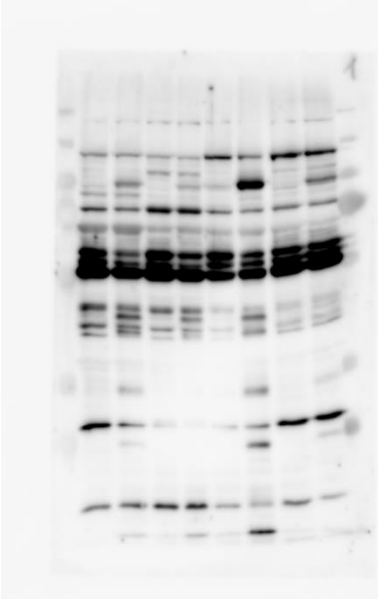

Parp

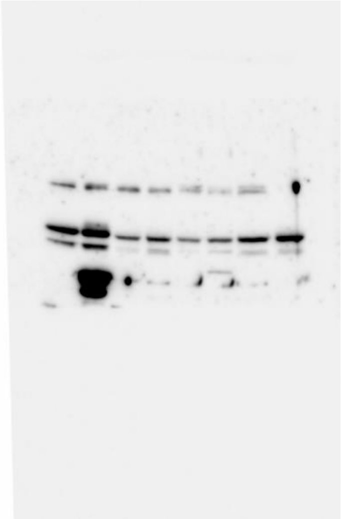

A20

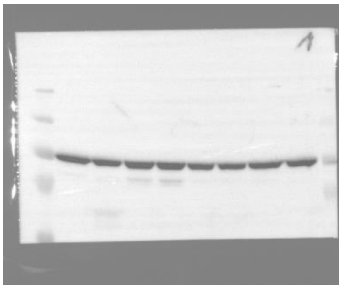

HSP90

Abbildung 2D Panc1

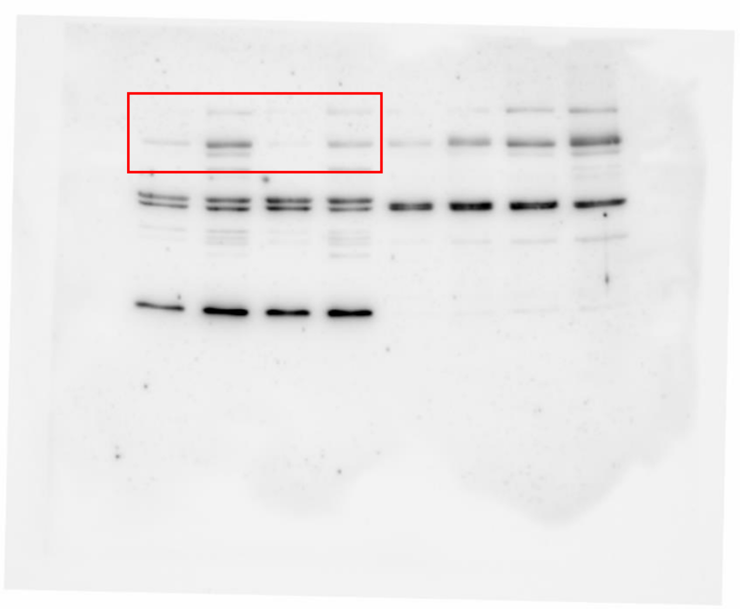

A20

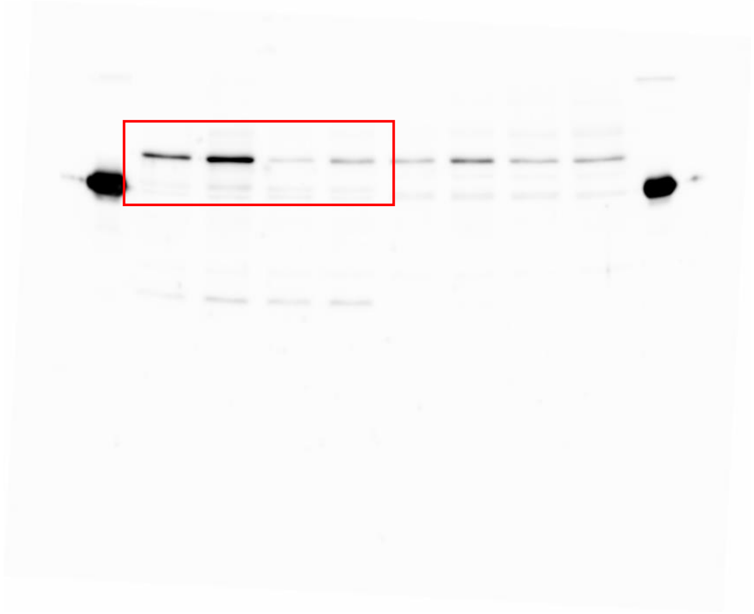

RelA

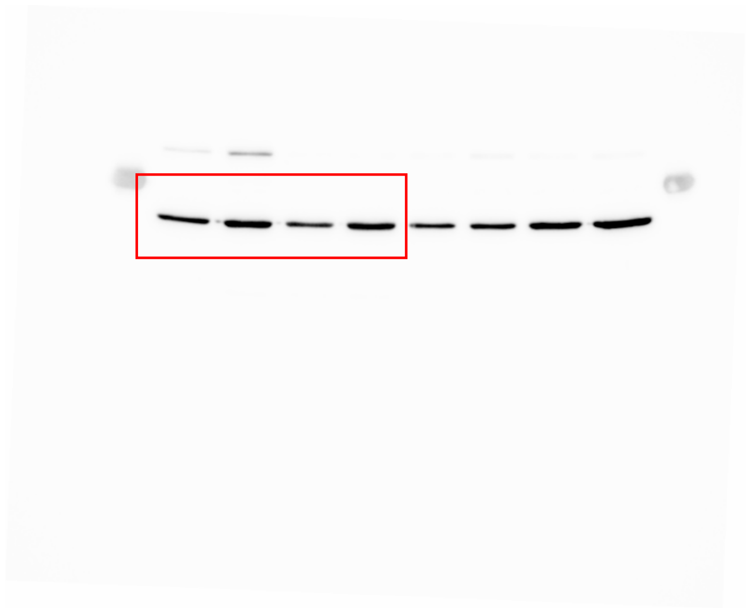

beta actin

Abbildung 2D PaTu8902

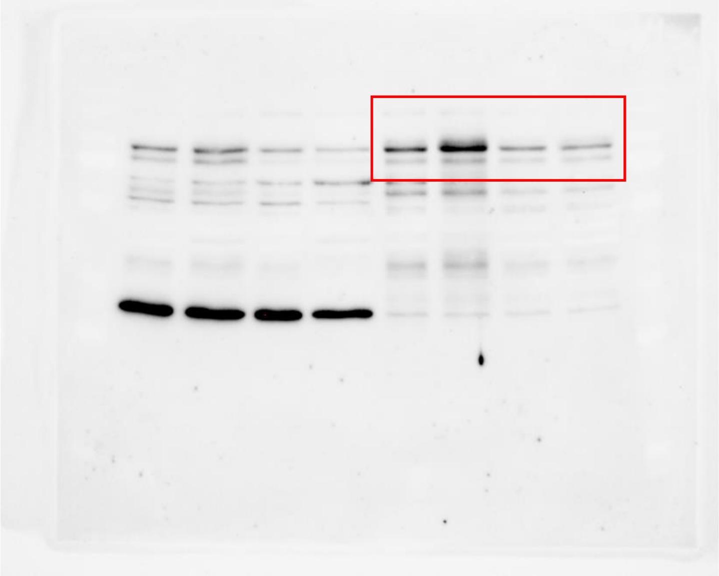

A20

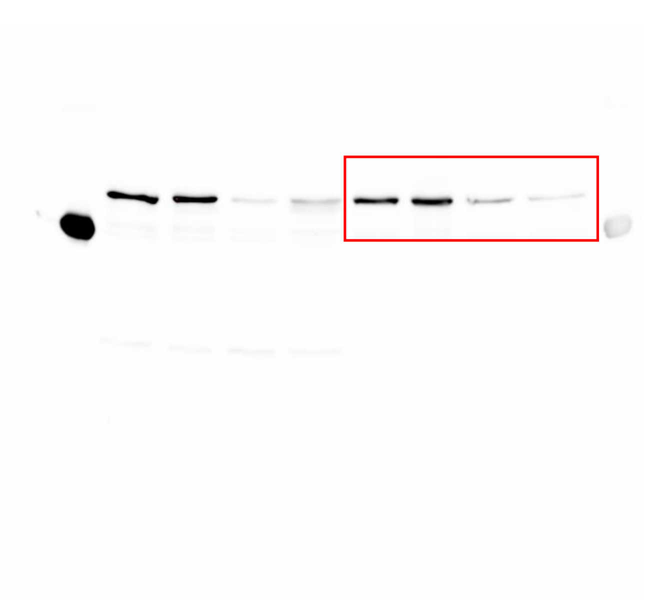

RelA

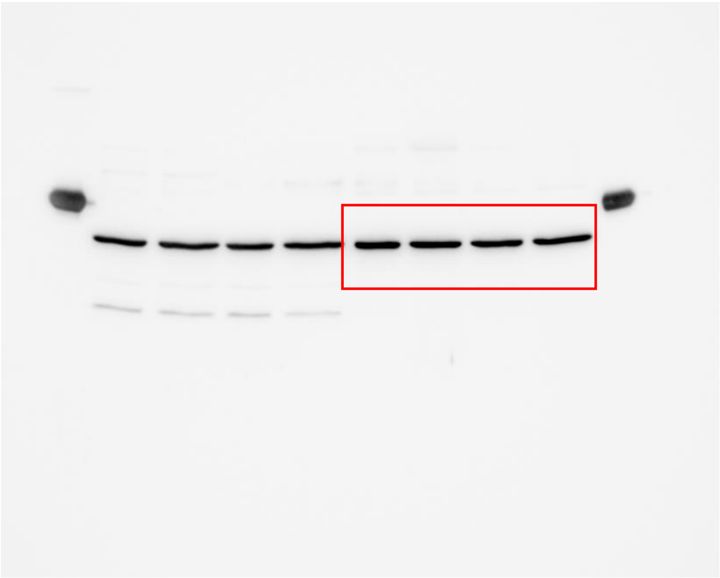

beta actin

Abbildung 5A Panc1

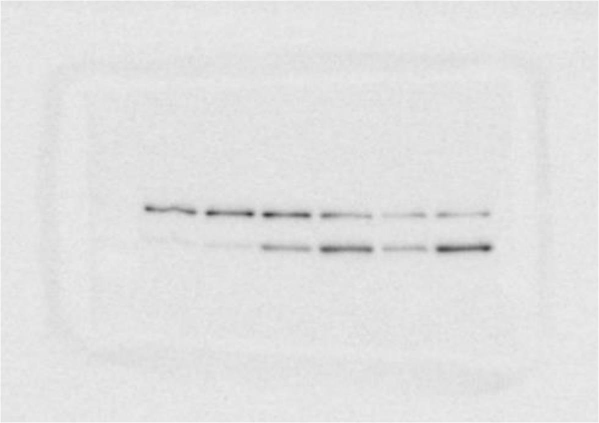

Parp

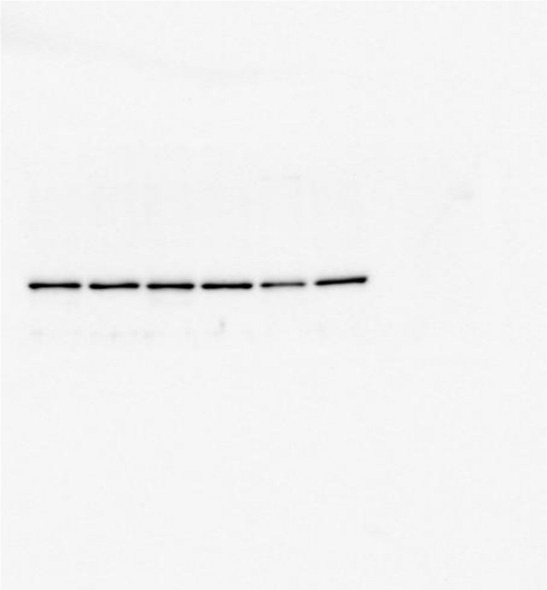

HSP90

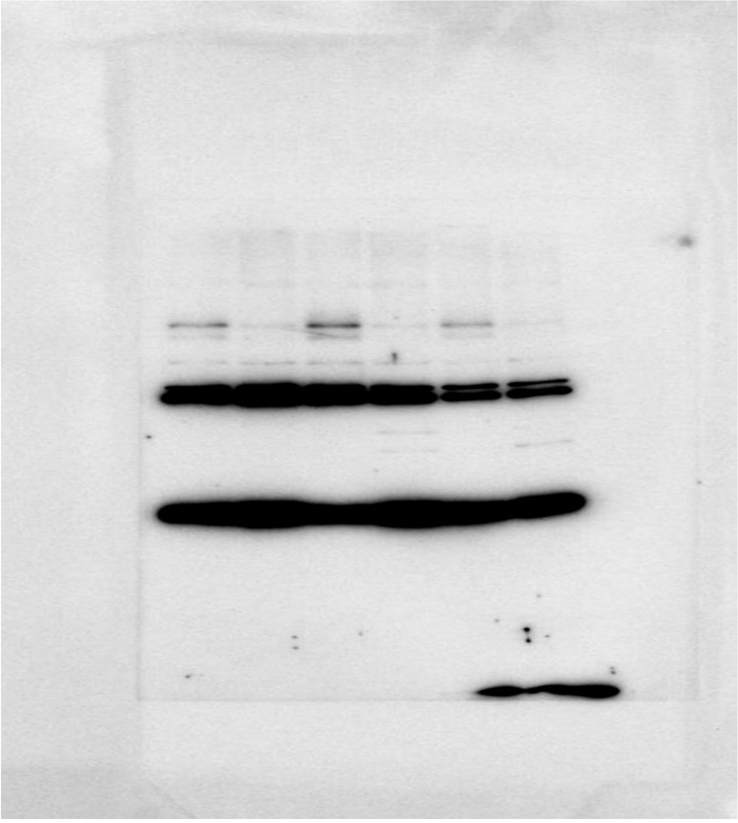

A20

Abbildung 5A Panc1

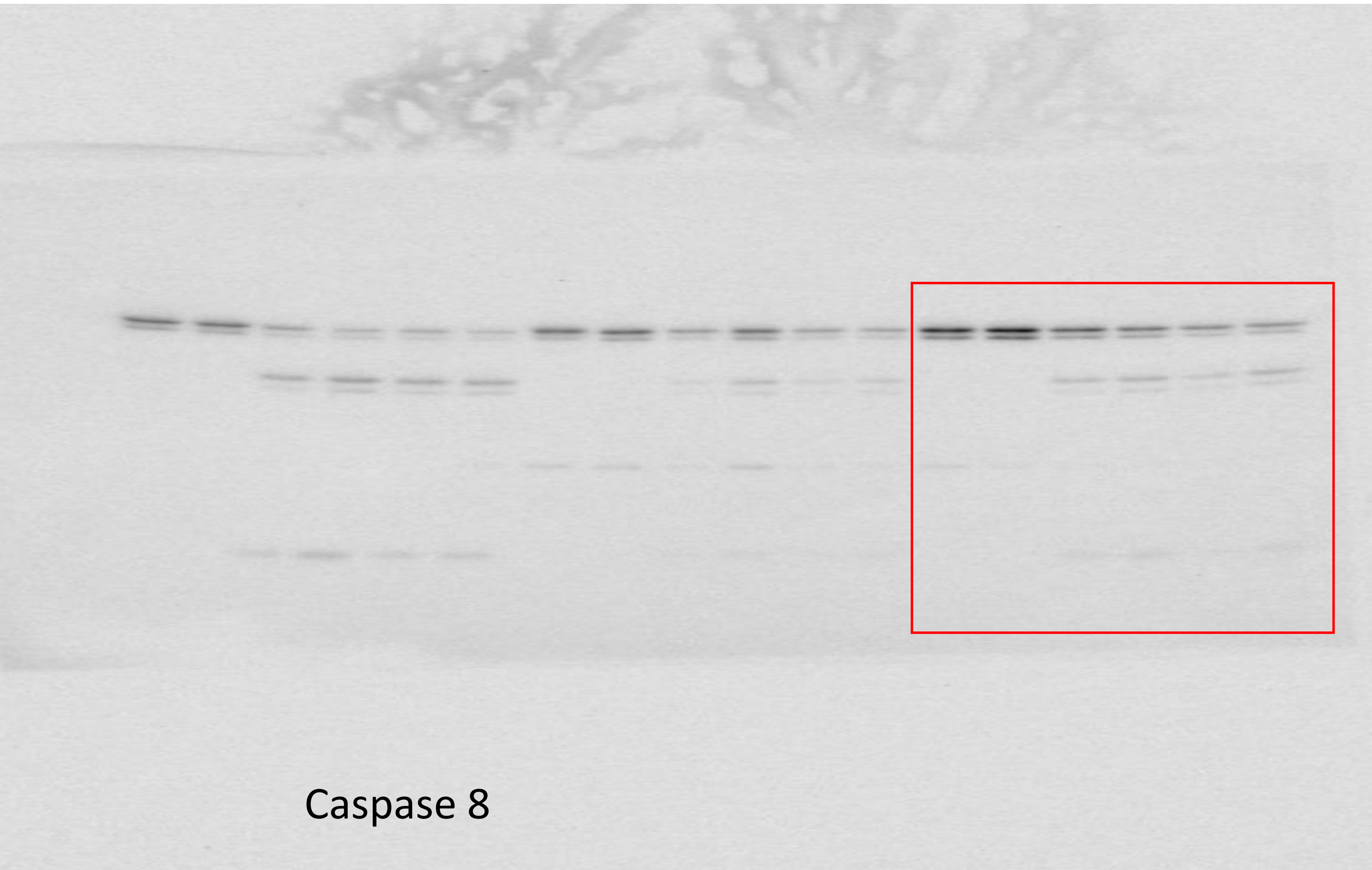


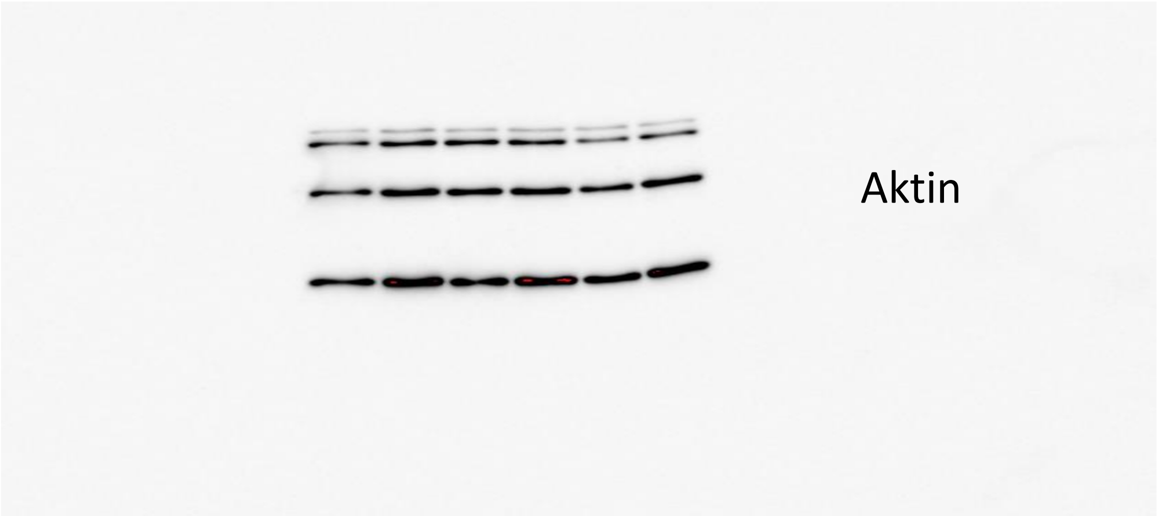

Abbildung 5A Patu8988t

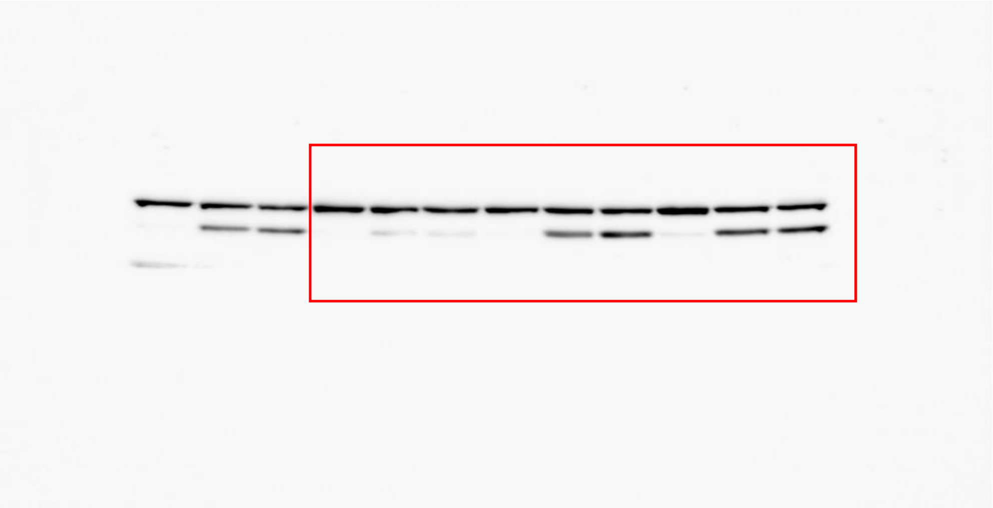

Parp

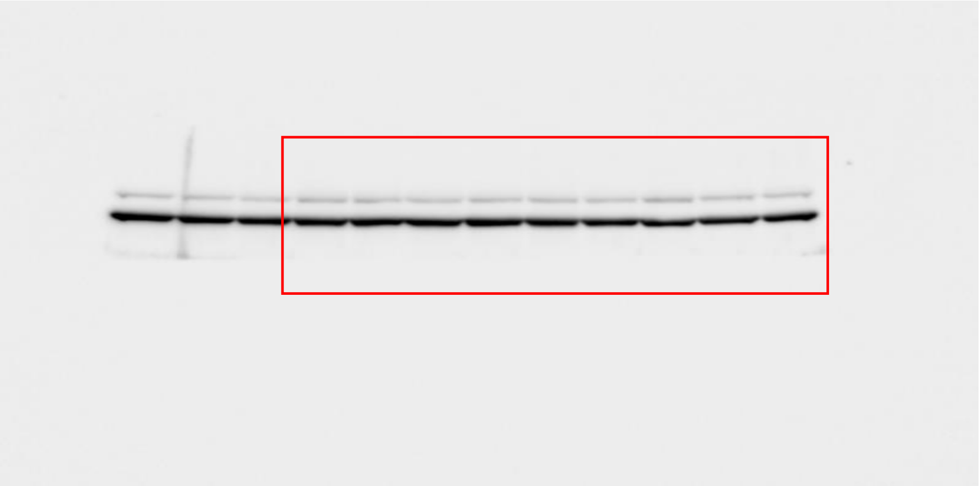

HSP90

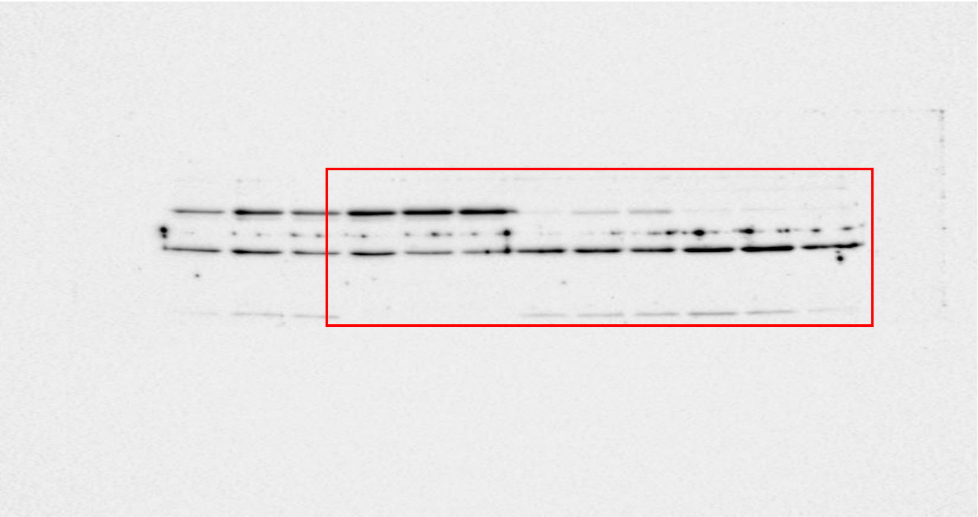

A20

Abbildung 5A Patu8988t

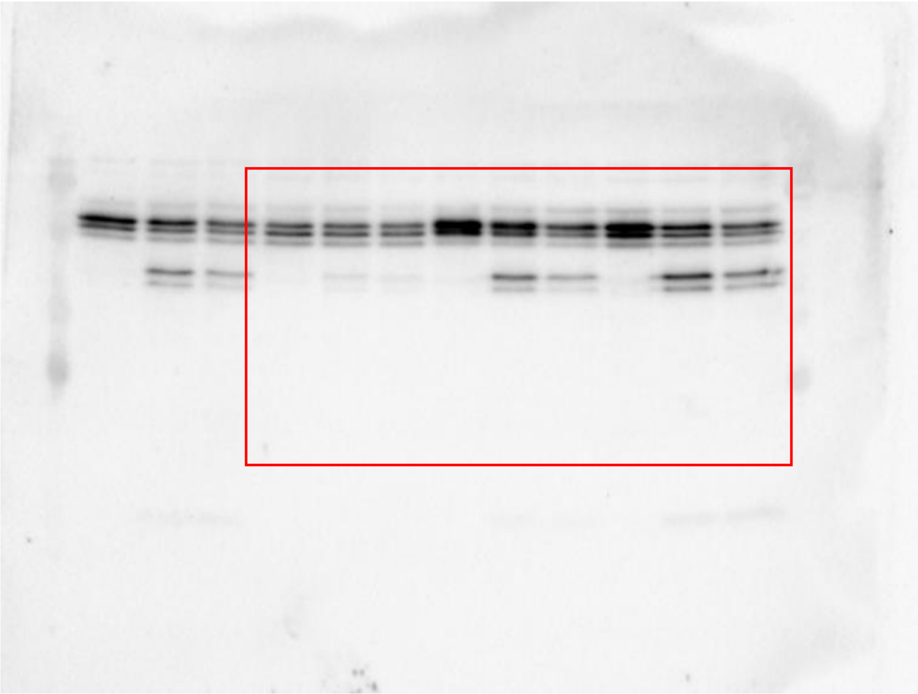

Caspase 8

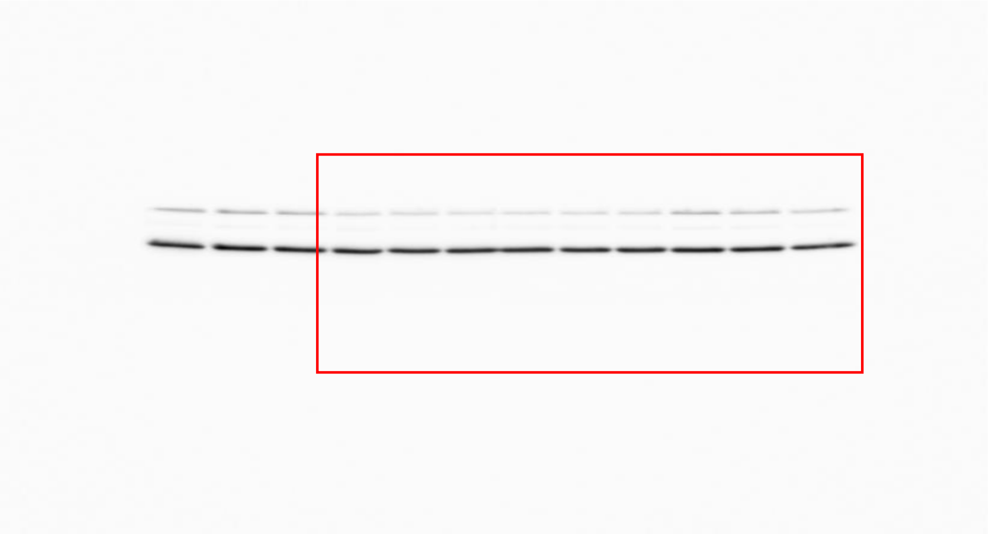

beta actin

Abbildung 7A Panc1

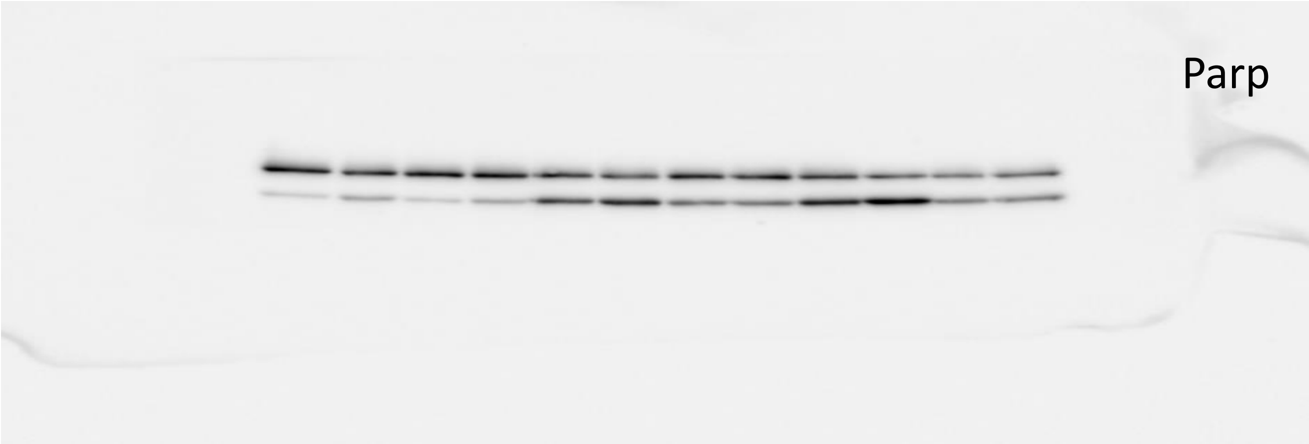

Parp

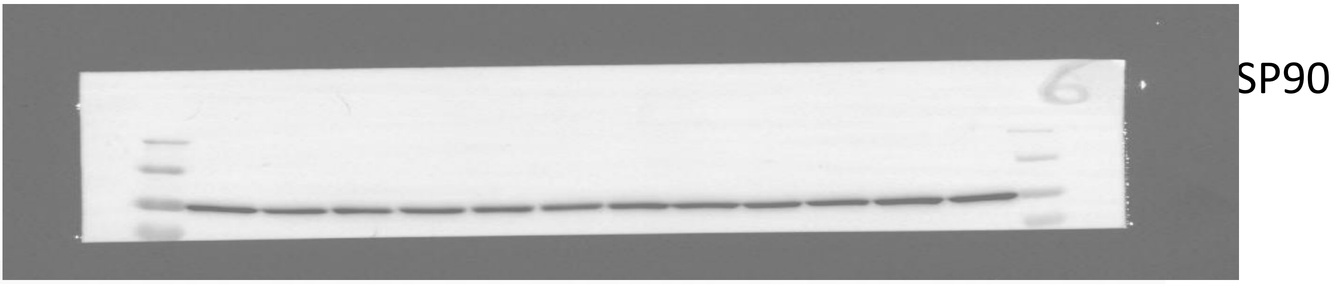

SP90

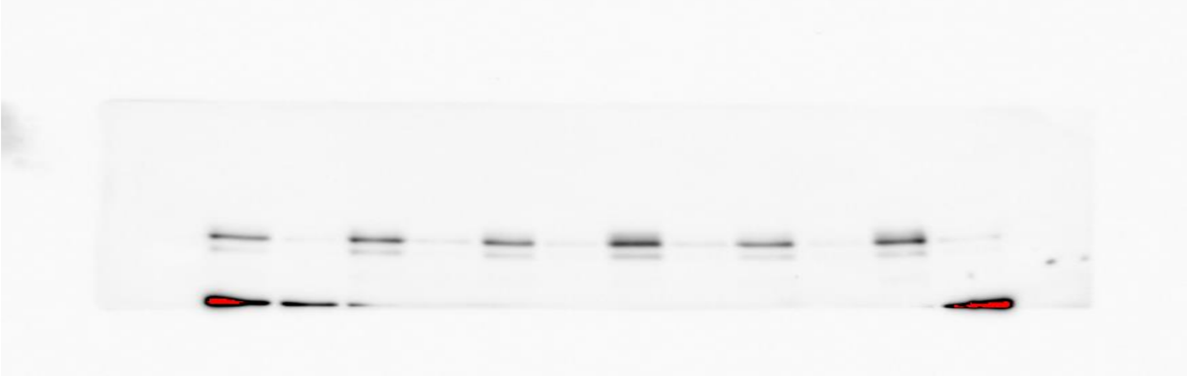

A20

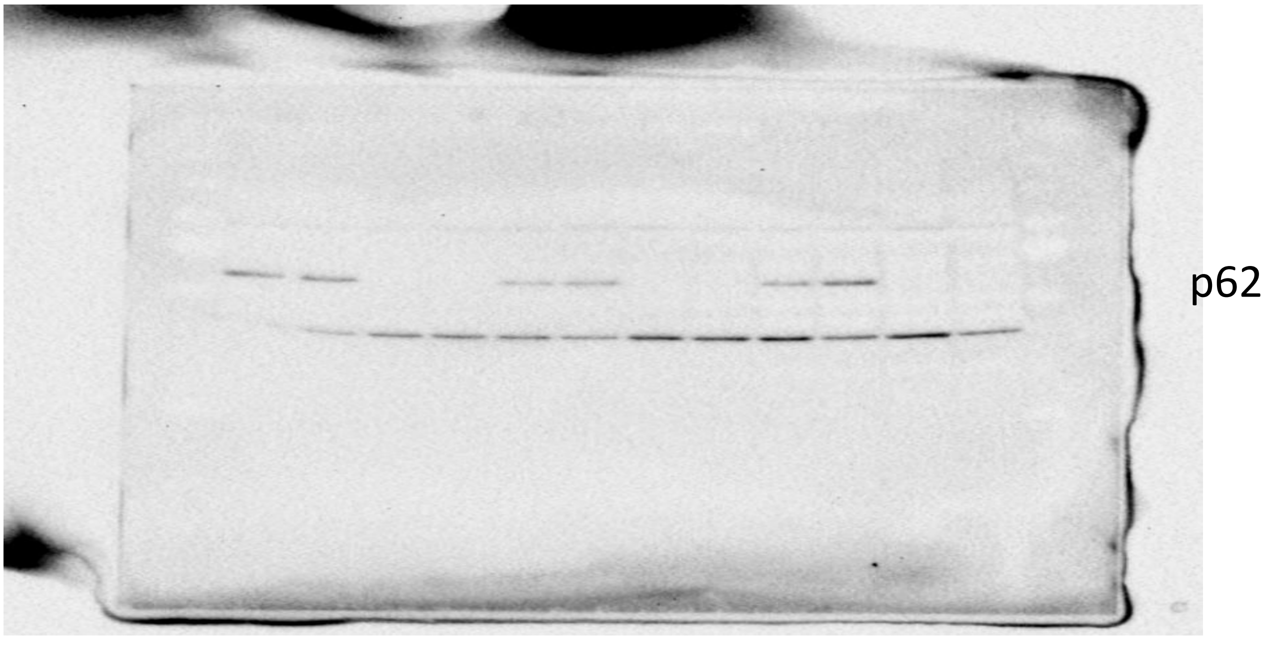

p62

Abbildung 7B PaTu8902

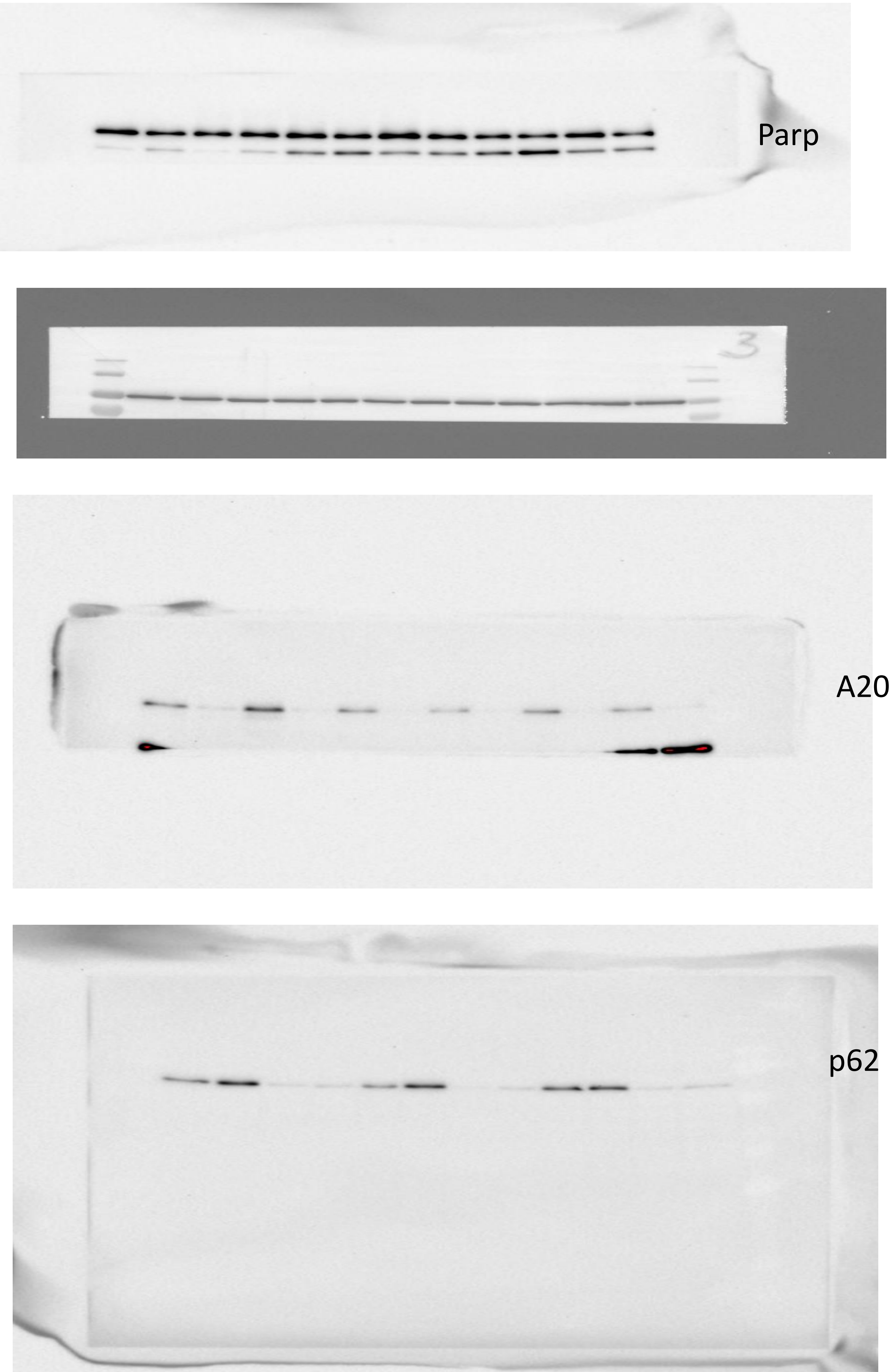

Supplement: Supplementary file 3 — Original Data File [file 41419_2022_5535_MOESM3_ESM.pdf]
